# Screening and transcriptomic profiling of tobacco growth-promoting arbuscular mycorrhizal fungi

**DOI:** 10.1080/15592324.2025.2467935

**Published:** 2025-02-19

**Authors:** Shuang-Lin Yang, Xiao-Xu Bi, Bo Huang, Ti-Yuan Xia, Li-Juan Deng, Xiao-Qin Luo, Yu Zhong, Yu-Ping Zhang, Ying-Ying Qian, Min Yin, Zhen Ren

**Affiliations:** a School of Agriculture and Life Sciences, Kunming University, Kunming, Yunnan, China; b College of Urban Construction Engineering, Guangzhou City polytechnic, Guangzhou, Guangdong, China; c China Tobacco Yunnan Industrial Co, Ltd, Kunming, Yunnan, China; d School of Medicine, Yunnan University, Kunming, Yunnan, China

**Keywords:** Tobacco, Arbuscular Mycorrhizal Fungi (AMF), growth promotion, RNA-Seq

## Abstract

Tobacco is a significant economic crop cultivated in various regions of China. Arbuscular mycorrhizal fungi (AMF) can establish a symbiotic relationship with tobacco and regulate its growth. However, the influences of indigenous AMF on the growth and development of tobacco and their symbiotic mechanisms remain unclear. In this study, a pot inoculation experiment was conducted, revealing that six inoculants - *Acaulospora bireticulata*(Ab), *Septoglomus viscosum*(Sv), *Funneliformis mosseae*(Fm), *Claroideoglomus etunicatum*(Ce), *Rhizophagus intraradices*(Ri), and the mixed inoculant (H) – all formed stable symbiotic relationships with tobacco. These inoculants were found to enhance the activities of SOD, POD, PPO, and PAL in tobacco leaves, increase chlorophyll content, IAA content， CTK content， soluble sugars, and proline levels while reducing malondialdehyde content. Notably, among these inoculants, Fm exhibited significantly higher mycorrhizal infection density, arbuscular abundance, and soil spore density in the root systems of tobacco plants compared to other treatments. Membership function analysis confirmed that Fm had the most pronounced growth-promoting effect on tobacco. The transcriptome analysis results of different treatments of CK and inoculation with Fm revealed that 3,903 genes were upregulated and 4,196 genes were downregulated in the roots and stems of tobacco. Enrichment analysis indicated that the majority of these genes were annotated in related pathways such as biological processes, molecular functions, and metabolism. Furthermore, differentially expressed genes associated with auxin, cytokinin, antioxidant enzymes, and carotenoids were significantly enriched in their respective pathways, potentially indirectly influencing the regulation of tobacco plant growth. This study provides a theoretical foundation for the development and application of AMF inoculants to enhance tobacco growth.

## 1. Introduction

Tobacco (*Nicotiana tabacum* L.) is an annual or limited perennial herbaceous plant of the genus *Nicotiana* in the Solanaceae family.^1^ China leads the world in tobacco production and planting areas, with tobacco serving as the main economic source for farmers in many regions of the country.^2^ As the tobacco planting area expands, the color, aroma, maturity, and chemical composition of tobacco leaves become the key indicators for evaluation.^3,4^ However, continuous cropping and insufficient cultivation management have significantly reduced the tobacco yield and quality. Although chemical fertilizers are commonly used to enhance the yield and quality, their irrational application and overuse result in the extended growth and the delayed maturity, adversely affecting the baking process, increasing the yield of inferior tobacco, and severely damaging soil quality and the environment.^5,6^ With the growing market demand for green tobacco, microbial fertilizers, which focus on microbial agents, have garnered widespread attention from domestic and international researchers. The excellent strain resources are fundamental for research and development of microbial fertilizers.

As a group of soil-dwelling microorganisms, Arbuscular Mycorrhizal Fungi (AMF) can establish reciprocal symbiosis with over 90% of terrestrial plant roots.^7^ Upon colonizing plant roots, AMF significantly enhance the plant growth and development, increase the water and nutrient uptake, improve the stress resistance, and support the biodiversity and nutrient cycling.^8^ The AMF inoculation has been shown to affect the tobacco growth. For example, Begum et al.^9^ reported that plant height, fresh stem biomass, and dry stem biomass of the *Glomus versiforme* group were 57.15%, 60.83%, and 75.73% higher, respectively, than those of the control group. Subhashini^10^ observed the increased plant height, leaf number, and rhizome dry weight in the *Glomus versiforme* group compared to the untreated control group. Liu et al.^11^ demonstrated that the inoculation with *Funneliformis mosseae* enhanced the root biomass, the leaf photosynthetic rate, and the antioxidase activity under various moisture conditions, significantly promoting the tobacco plant growth. Overall, the AMF inoculation improved the tobacco survival, growth, and biomass.

RNA-Seq is an advanced technology for exploring the gene pathways and mechanisms with high sensitivity, including the detection of splicing isoforms and somatic mutations.^12^ Recently, RNA-Seq has been extensively applied to analyze the whole transcriptome of plants colonized by AMF and non-AMF, providing insights into the effects of AMF on the plant gene expression.^13^ Ma et al.^14^ reported that the mycorrhizal watermelon exhibited significantly better development than the non-mycorrhizal treatment, with RNA-Seq identifying 2259 differentially expressed genes (DEGs) in mycorrhizal watermelon roots, primarily involved in photosynthesis, chlorophyll biosynthesis, hormone biosynthesis, and nutrient transport. Ran et al.^15^ discovered 585 DEGs in the roots of mycorrhizal *Panax quinquefolius* L., compared to the CK, which were significantly enriched in pathways such as the “protein processing in the endoplasmic reticulum”, the “drug metabolism-cytochrome P450”, the “plant-pathogen interactions”, the “glutathione metabolism”, and the “plant hormone signal transduction”. Jing et al.^16^ demonstrated through RNA-Seq that AMF promoted the lateral root development in apple plants by affecting the sugar metabolism, the fatty acid metabolism, and the hormone metabolism, and also enhanced the above-ground growth by influencing the expression of hormone metabolism and cellular morphogenesis-related genes.

Previous studies on the symbiosis between AMF and tobacco have primarily focused on the diversity of AMF in tobacco roots and the effects of one or two AMF species on tobacco growth, development, and stress resistance.^9–11^ However, research on the effects of multiple AMF species on tobacco growth and the impact of AMF on the expression of genes related to growth promotion remains limited. Therefore, this study adopted K326, a primary tobacco cultivar in Yunnan, China, as the research subject to investigate the effects of the pre-inoculation with *Acaulospora bireticulata*, *Septoglomus viscosum*, *Funneliformis mosseae*, *Claroideoglomus etunicatum*, and *Rhizophagus intraradices*, and five mixed microbial agents. A pot inoculation experiment identified the microbial agents with the most effective growth-promoting effects. RNA-Seq was employed to explore the impact of these microbial agents on genes associated with tobacco growth promotion. This result may provide the valuable references and the strain resources for the development and application of tobacco growth-promoting microbial agents.

## 2. Materials and methods

### 2.1. Tobacco, AMF, and cultivation substrate

Tobacco K326 seeds were supplied by Yunnan Tobacco Company and planted in March 2023 until they developed 1–3 true leaves for subsequent application. The indigenous AMF microbial agents including *A. bireticulata*, *S. viscosum*, *F. mosseae*, *C. etunicatum*, and *R. intraradices* were the dominant AMF isolated from the tobacco root zone in central Yunnan, China, and propagated through corn roots after single spore identification. The cultivation soil was sourced from the Guangwu Mountain of Kunming College (E102°47′42.72″, N24°58′25.32″), with the cultivation substrate consisting of a sterilized mixture of soil, perlite, and vermiculite in a 2:1:1 ratio. The physical and chemical properties of the soil were pH of 6.50, organic matter of 30.88 g/kg, total nitrogen of 3.20 g/kg, total phosphorus of 2.50 g/kg, and total potassium of 6.22 g/kg.

### 2.2. Experimental design and indicator determination

#### 2.2.1. Pot experiment

The one-way ANOVA design was employed with seven groups: the control group (CK), the inoculation with *A. bireticulata* (Ab), *S. viscosum* (Sv), *F. mosseae* (Fm), *C. etunicatum* (Ce), *R. intraradices* (Ri), and the mixed microbial agent (H). Each group consisted of 12 replicates, resulting in 84 pots. Each pot contained 1.5 kg of cultivation substrate, with the additional 100 g of the AMF microbial agent (approximately 600 spores) applied (excluding CK), and was then covered with 0.5 kg of cultivation substrate after transplanting the tobacco seedlings. After 180 d of growth, the measurements were taken for the AMF colonization rate, the spore density in soil, and the physiological indicators of tobacco growth. Subsequently, the membership function analysis was performed to identify the AMF microbial agent most effective in promoting tobacco growth based on physiological measurements.

##### 2.2.1.1. Determination of AMF infection and spore density in soil

The trypan blue staining method was employed to assess AMF infection, while “Mycocalc” software^17^ was utilized to calculate various mycorrhizal colonization rate parameters. The wet sieve sucrose centrifugation method was used to determine the spore density in the soil.

##### 2.2.1.2. Determination of tobacco growth physiological indicators

The fresh weight, stem diameter, root length, and height of tobacco plants were measured using the conventional methods. The leaf area was determined using a leaf area meter (CI–202). In accordance with the methodologies reported in the literature, the samples were subjected to a series of treatments as described below. Chlorophyll extraction was performed using an ethanol-acetone mixed solvent (1:1 ratio).^18^ The activity of superoxide dismutase (SOD) in leaves was assessed via the nitroblue tetrazolium reduction method.^19^ Polyphenol oxidase (PPO) activity was quantified using the catechol method.^19^ Peroxidase (POD) activity was determined through the guaiacol colorimetric method.^20^ Phenylalanine ammonia-lyase (PAL) activity was measured by the mercaptoethanol method.^20^ Soluble sugar content was evaluated using the phenol-sulfuric acid method.^21^ Proline content was determined via the ninhydrin colorimetric method.^22^ Malondialdehyde (MDA) content was measured using the thiobarbituric acid method.^23^ Total phenolics (TP) were quantified by the Folin-phenol method.^24^ Flavonoid content was determined through visible spectrophotometry.^24^ Following these standardized procedures, all samples were analyzed using a C-7100 UV-Vis spectrophotometer (Peakin Instrument Shanghai Co., Ltd.). Auxin (IAA) and cytokinin (CTK) contents were pre-treated with ELISA kits (Shanghai Jin Ning Biotechnology Co., Ltd.) and subsequently measured using a SPECTRAMAX190 multi-function microplate reader (Thermo Fisher Scientific).

##### 2.2.1.3. Evaluation by the membership function method

The membership function method was employed to comprehensively evaluate the growth-promoting effects of different microbial agents, allowing for the selection of the AMF type most effective in promoting the tobacco growth. The calculation formulas were as follows: F1 (Xi) = (Xi – Xmin)/(Xmax – Xmin) and F2 (Xi) = 1– (Xi – Xmin)/(Xmax – Xmin), where X is the value of the ith indicator, Xmin is the minimum value of the ith indicator, and Xmax is the maximum value of the ith indicator. If the indicator was inversely related to the tobacco growth effect, F2 was used to calculate the membership function value.

#### 2.2.2. RNA-Seq

The CK group and AMF – treated tobacco rhizomes demonstrating the best growth promotion effect in the pot experiment were selected. These samples were treated with liquid nitrogen for 30 min and then stored at −80°C for the subsequent RNA-Seq and qRT-PCR validation. Each group consisted of four replicates.

##### 2.2.2.1. RNA extraction and library construction

The total RNA was extracted from each treated tobacco rhizome using the TRIzol method.^25^ The concentration of the extracted RNA was measured using the ND5000 ultra-micro UV – visible spectrophotometer, while the RNA integrity and the DNA purity were assessed using 1.0% agarose gel electrophoresis. After passing these quality test, RNA-Seq was conducted by Shanghai Major Biotechnology Co. Ltd. The qualified RNA was enriched for mRNA using the oligo (dT) magnetic beads, fragmented using fragmentation buffer, synthesized into cDNA, screened for appropriate fragments using AMPure XP beads, and amplified by PCR to construct the final library. Sequencing was performed on the Novaseq 6000 platform, producing the raw data from which the clean reads were obtained after quality control.

##### 2.2.2.2. Sequencing data quality control

The Illumina MiSeq platform converted the image signals from sequencing into the raw data stored in fastq format. The quality control of the raw data was conducted using the software fastx_toolkit_0.0.14 to obtain the high-quality clean data. The low-quality 3’-end sequences were trimmed, retaining only the reads over 50 bp for further analysis. The quality control was performed using SeqPrep (https://github.com/jstjohn/SeqPrep.) and Sickle (https://github.com/najoshi/sickle).

##### 2.2.2.3. Sequence alignment

The reference gene source was Nicotiana_tabacum, using the reference genome version GCF_000715135.1, accessible at https://www.ncbi.nlm.nih.gov/genome/?term=txid4097[orgn]. The Sequence alignment was performed using the TopHat2 (http://ccb.jhu.edu/software/tophat/index.shtml.) and HISAT2 (http://ccb.jhu.edu/software/hisat2/index.shtml) databases. Assembly evaluation was conducted using Cufflinks (http://cole-trapnell-lab.github.io/cufflinks/.) and StringTie (http://ccb.jhu.edu/software/stringtie/). These tools were used to compare with the original genome annotation, identify previously unannotated transcribed regions, and discover new transcripts and genes, thereby enhancing the original genome annotation information.

##### 2.2.2.4. Analysis of DEGs

The raw counts were analyzed using DESeq2 to identify DEGs by comparing the expression of tobacco rhizome genes between the two groups. The screening criteria were set to *p* < 0.05, and |log2FC|≥1. The sequences of the identified DEGs were annotated for the GO and KEGG pathways using Blast2GO and KOBAS, respectively, to determine the primary biological functions and pathways of the DEGs.

##### 2.2.2.5. Verification by qRT – PCR

To verify the accuracy of the RNA-Seq results, seven key DEGs associated with growth promotion, antioxidase activity, and terpenoid skeleton biosynthesis were selected for the qRT – PCR validation using actin as the internal reference gene. The specific primers were designed using the web version of NCBI Primer BLAST (Table 1), and the gene expression was measured using the fluorescence quantitative PCR (JF-PCR-16, Germany). The reaction conditions were as follows: 95°C for 3 min, followed by 39 cycles of 95°C for 10 s and 58°C for 30 s, with the rapid increase to 95°C to collect the fluorescence, and the final melting curve analysis at 95°C for 15 s. The relative gene expression was calculated using the 2^−△△CT^ method.

**Table 1.** Primer sequences used for qRT – pcr.

Gene	Gene description	Upstream primer ^(5’-3’)^	Downstream primer ^(5’-3’)^
actin	/	CGGAATCCACGAGACTACATAC	GGGAAGCCAAGATAGAGC
*LOC107765489*	peroxidase 3-like	CCATCTCTTGCAGCTGCCTT	ACAGATCCATCACAACCCCTG
*LOC107777482*	peroxidase 44-like	AGCTCATACAGTTGGAGTCGT	GGATCCATTGTGGGGTCAGG
*LOC107783920*	auxin-induced protein 15A-like	CGGCGATCCCTGGCAAC	GCGCTGCAAAGAAAAACTTCC
*LOC107803574*	probable indole-3-acetic acid-amido synthetase GH3.1	GATGCCCACAATTGCGGATG	ACAGATTCATCACGGGCTGG
*LOC107780689*	probable indole-3-acetic acid-amido synthetase GH3.1	CAACTGCTCCTCACTGGACC	AGCTAAGGCCTAATGGAAAATGT
*LOC107806551*	farnesyl pyrophosphate synthase 1-like	ATTGGTGGCTGGCGCTTAC	CCAGCGCCGTATTTCTCTGA
*LOC107822513*	geranylgeranyl pyrophosphate synthase 7, chloroplastic-like	CGGGGCAGATTGTGGACATA	TCCCCCAATTATTGCCCCAC

### 2.3. Statistical analysis

The data organization and preliminary calculations were performed using Microsoft Excel 2016. The statistical analyses were conducted with SPSS software, version 26.0, utilizing the one-way Analysis of Variance (ANOVA) to assess the significance of observed differences among groups. The graphs and visualizations were created using Origin software version 2021. The results were presented as the mean ± standard deviation (SD). In the graphical representations, the groups with the same letter were considered not significantly different, while the groups with different letters indicated the statistically significant difference at the 0.05 level.

## 3. Results and analysis

### 3.1. Effects of inoculation with AMF on tobacco root mycorrhizal infection and spore density

Table 2 presents the results of the root infection and rhizospheric spore density in soil for tobacco inoculated with different AMF. These findings indicated that the root colonization rate of tobacco inoculated with AMF was significantly higher than that of the CK group after 180 d of growth, suggesting the successful invasion and symbiosis establishment by various AMF. Among the AMF groups, Fm exhibited the highest level of root AMF infection, with the colonization rate of 100%, the infection intensity of 48.67%, and the arbuscular abundance of 26.91%. Additionally, the rhizospheric spore density in soil was the highest in the Fm group, with 343.33 AMF spores per 25 g of soil. Therefore, Fm demonstrated the superior symbiotic establishment with tobacco roots.

**Table 2.** Effects of inoculation with AMF on tobacco root mycorrhizal infection and spore density.

	CK	Ab	Ce	Sv	Fm	Ri	H
Infection rate (%)	8.89 ± 1.11^e^	26.43 ± 1.00^d^	48.3 ± 1.21^c^	92.22 ± 4.01^a^	100 ± 0^a^	81.11 ± 4.44^b^	52.03 ± 3.93^c^
Root mycorrhizal infection density (%)	0.68 ± 0.04^e^	7.94 ± 0.45^de^	15.68 ± 0.66^cd^	35.87 ± 3.35^b^	48.67 ± 7.61^a^	22.88 ± 4.78^c^	20.33 ± 0.66^c^
Arbuscular abundance (%)	0.07 ± 0.03^d^	7.9 ± 0.40^cd^	14.82 ± 1.12^bc^	20.34 ± 3.54^b^	36.91 ± 6.16^a^	14.08 ± 4.99^bc^	10.39 ± 0.10^bcd^
Spore density (spore/25 g)	56 ± 3.04^c^	245 ± 20.57^b^	216 ± 8.44^b^	241 ± 20.36^b^	343.33 ± 19.28^a^	259 ± 25.46^b^	218 ± 13.78^b^

Values in the table are expressed as mean ± standard error (n = 3); different lowercase letters in the same row and the same treatment group indicate significant differences (p < 0.05).

### 3.2. Effects of inoculation with AMF on tobacco growth

#### 3.2.1. Effects of inoculation with AMF on tobacco growth

Table 3 indicates that both single and mixed microbial agents could enhance the tobacco growth, while the growth promotion effects of different AMF varied significantly. Compared to other AMF groups, the Fm and Sv groups significantly promoted tobacco growth. Specifically, the Fm group showed increases of 91.07% in plant height, 40.36% in root length, 16.00% in leaf area, 18.28% in stem diameter, 64.69% in aboveground fresh weight, 54.85% in belowground fresh weight, and 59.17% in total biomass compared to the CK group. Similarly, the Sv group demonstrated the increases of 84.01% in plant height, 46.01% in root length, 18.79% in leaf area, 20.99% in stem diameter, 72.24% in aboveground fresh weight, 37.62% in belowground fresh weight, and 52.79 in total biomass.

**Table 3.** Effects of inoculation with AMF on tobacco growth.

	CK	Ab	Ce	Sv	Fm	Ri	H
Plant height (cm)	32.15 ± 4.053^d^	51.36 ± 2.93^abc^	41.69 ± 7.08^cd^	59.16 ± 2.89^ab^	61.43 ± 0.34^a^	49.18 ± 2.01^bc^	55.41 ± 1.10^ab^
Root length (cm)	26.19 ± 1.27^b^	33.46 ± 0.91^a^	35.28 ± 2.02^a^	38.24 ± 1.56^a^	36.76 ± 1.99^a^	35.46 ± 3.44^a^	26.12 ± 0.37^b^
Leaf area (cm^2^)	586.6 ± 10.54^c^	682.71 ± 30.89^ab^	603.25 ± 0.72^bc^	696.82 ± 26.04^a^	680.47 ± 35.59^ab^	587.6 ± 31.05^c^	637.19 ± 20.01^abc^
Stem diameter (cm)	4.43 ± 0.09^b^	4.56 ± 0.28^b^	4.53 ± 0.17^b^	5.36 ± 0.09^a^	5.24 ± 0.13^a^	5.2833 ± 0.09^a^	3.62 ± 0.31^c^
Above-ground biomass (g)	227.73 ± 7.29^c^	365.58 ± 32.22^ab^	296.41 ± 27.22^bc^	392.24 ± 3.91^a^	375.05 ± 29.35^ab^	301.13 ± 27.72^bc^	316.37 ± 30.42^ab^
Below-ground biomass (g)	291.77 ± 21.17^b^	323.1 ± 2.28^b^	279.28 ± 18.71^b^	401.52 ± 4.26^a^	451.83 ± 21.57^a^	397.15 ± 40.25^a^	308.96 ± 5.05^b^
Total biomass (g)	519.5 ± 20.25^d^	688.68 ± 33.64^b^	575.69 ± 8.93^cd^	793.76 ± 6.64^a^	826.88 ± 34.59^a^	698.28 ± 13.86^b^	625.33 ± 26.45^bc^

Values in the table are expressed as mean ± standard error (n = 3); different lowercase letters in the same row and the same treatment group indicate significant differences (p < 0.05).

#### 3.2.2. Effects of inoculation with AMF on activity of antioxidase in tobacco

**Figure 1.**
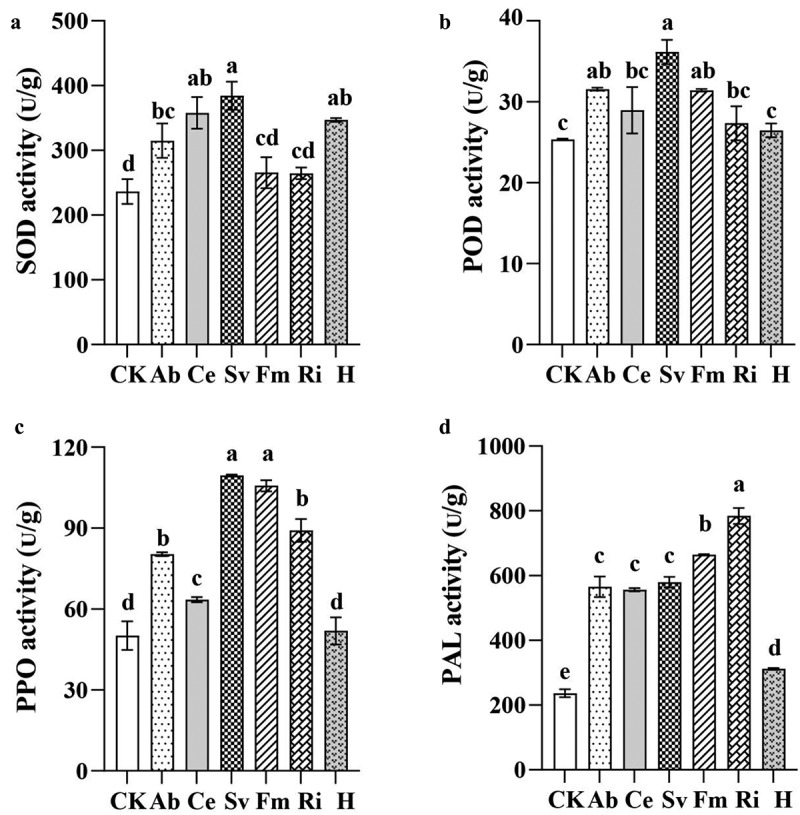
Effects of inoculation with AMF on activity of antioxidase in tobacco.

Figure 1 illustrates that the AMF inoculation significantly affected the activities of SOD, POD, PPO, and PAL in tobacco. Compared to the CK group, the activities and contents of these enzymes were notably higher in the AMF groups, whereas the extent of increase varied among the microbial agents. Specifically, the Sv group exhibited the highest SOD, POD, and PPO activities, with increases of 56.71%, 42.59%, and 118.34%, respectively. The Ri group demonstrated the highest PAL activity, with the increase of 231.49%, followed by the Fm group with the increase of 189.28%.

#### 3.2.3. Impact of AMF inoculation on phytohormone levels in tobacco leaves

**Figure 2.**
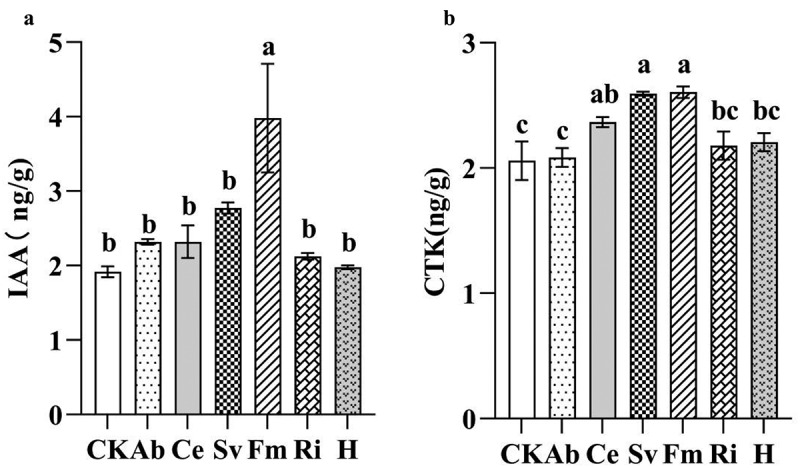
Effects of inoculation with AMF on tobacco IAA and CTK contents.

As illustrated in Figure 2, the levels of IAA and CTK in tobacco leaves following AMF inoculation exhibited increases, with varying effects depending on the specific AMF treatment. Compared to the control (CK), the IAA content in Fm-treated samples increased significantly by 107.62%, while other treatments did not reach statistical significance. Meanwhile, compared to CK, the CTK content in Fm-, Sv-, and Ce-treated samples increased significantly by 26.58%, 25.96%, and 14.95%, respectively. Other treatments did not achieve statistical significance.

#### 3.2.4. Effects of inoculation with AMF on tobacco chlorophyll and osmoregulatory substance content

**Figure 3.**
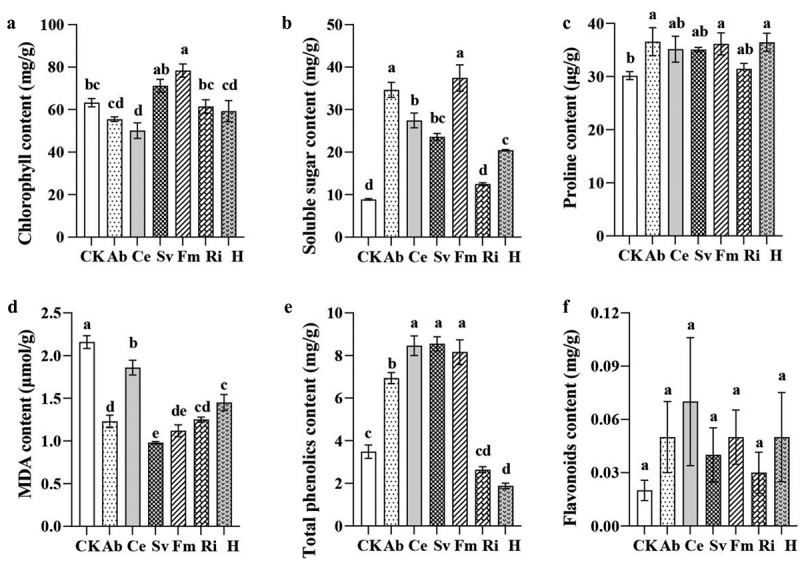
Effects of inoculation with AMF on tobacco chlorophyll and other osmoregulatory substance contents.

Figure 3 illustrates the effects of the inoculation with different AMF on the tobacco chlorophyll and osmoregulatory substance contents. The Fm group had the highest chlorophyll and soluble sugar contents in the leaves, with the significant increases of 23.83% and 322.10%, respectively, compared to the CK group. The analysis of proline and MDA content revealed that various AMF could increase the proline content and decrease the MDA content in tobacco. The proline content was significantly higher in the tobacco plants inoculated with Ab, H, or Fm, with the increases of 21.22%, 20.86%, and 19.86%, respectively. All AMF significantly reduced the MDA content, with the Sv and Fm groups demonstrating the greatest reductions of 54.63% and 48.15%, respectively. The total phenol content was significantly increased by the inoculation with Sv, Ce, Fm, and Ab, with the increases of 144.99%, 142.41%, 133.81%, and 98.85%, respectively. Although all six AMF groups increased the flavonoid contents compared to the CK group, the differences were not statistically significant.

### 3.3. Evaluation of the growth promotion effect of AMF microbial agent on tobacco

The membership function method was employed to evaluate the growth-promoting effects of the different AMF microbial agents on tobacco growth (Table 4). The results indicated that both the single and mixed microbial agents could enhance the tobacco growth, while the effects varied significantly among different AMF. The Fm group achieved the highest mean membership function value (0.87), suggesting that Fm was the most effective AMF microbial agent for promoting the tobacco growth, particularly in terms of plant height, below-ground biomass, total biomass, chlorophyll, IAA, CTK and soluble sugar. Overall, the growth promotion effects of the different AMF were summarized as Fm > Sv > Ab > Ce > Ri > H > CK.

**Table 4.** Inoculation with AMF on the membership function of various indicators of tobacco.

Indices	CK	Ab	Ce	Sv	Fm	Ri	H
Plant height	0.00	0.66	0.33	0.92	1.00	0.58	0.79
root length	0.01	0.61	0.76	1.00	0.88	0.77	0.00
Leaf area	0.00	0.87	0.15	1.00	0.85	0.01	0.46
Stem diameter	0.47	0.54	0.52	1.00	0.93	0.96	0.00
Above-ground biomass	0.00	0.84	0.42	1.00	0.90	0.45	0.54
Below-ground biomass	0.07	0.25	0.00	0.71	1.00	0.68	0.17
Total biomass	0.00	0.55	0.18	0.89	1.00	0.58	0.34
SOD activity	0.00	0.53	0.82	0.91	0.33	0.19	0.74
POD activity	0.00	0.57	0.33	1.00	0.56	0.19	0.10
PPO activity	0.00	0.51	0.22	1.00	0.94	0.66	0.03
PAL activity	0.00	0.60	0.58	0.61	0.82	1.00	0.14
IAA content	0.00	0.20	0.19	0.41	1.00	0.10	0.03
CTK content	0.00	0.05	0.56	0.98	1.00	0.22	0.27
Chlorophyll content	0.47	0.19	0.00	0.75	1.00	0.40	0.32
Soluble sugar content	0.00	0.90	0.65	0.52	1.00	0.13	0.40
Proline content	0.00	1.00	0.78	0.77	0.94	0.20	0.98
MDA content	0.00	0.79	0.25	1.00	0.88	0.77	0.60
Total phenol content	0.24	0.76	0.99	1.00	0.94	0.11	0.00
Flavonoids content	0.00	0.60	1.00	0.40	0.60	0.20	0.60
Average value	0.07	0.58	0.46	0.83	0.87	0.43	0.34
Sort by promotion effect	7	3	4	2	1	5	6

### 3.4. RNA-Seq reveals changes in the expression of key genes in tobacco plants inoculated with Fm

#### 3.4.1. Evaluation and splicing of sequencing data

To further elucidate the changes in the expression of the tobacco genes related to the growth promotion in the Fm group, we performed RNA-Seq on tobacco rhizomes from both the CK and Fm groups (Table 5). The average of 44,297,705.75 raw reads was obtained across the eight samples, with the maximum of 46,867,074 and the minimum of 41,980,420. After the quality control, the CK and Fm groups yielded the averages of 43,295,425.5 and 43,986,041 clean reads, respectively. The proportions of Q20% and Q30% were more than 97.08% and 95.05%, respectively. The average GC content percentages were similar for both groups, at 43.34% and 43.91%, respectively, and the percentage of unique mapping ranged from 78.64% to 89.75%. Hence, the RNA-Seq results were reliable and suitable for further bioinformatic analysis.

**Table 5.** RNA-Seq data statistics table.

Sample	Raw reads	Clean reads	Q20%)	Q30%)	GC content (%)	Multiple mapped (%)	Uniquely mapped (%)
CK_1	43098692	42365560	97.28	95.36	43.79	3.53%	89.28%
CK_2	44932552	44217640	97.08	95.05	43.56	3.55%	89.75%
CK_3	43180894	42511412	97.31	95.42	44.02	3.45%	87.09%
CK_4	44845236	44087090	97.12	95.12	44.26	3.31%	85.67%
Fm_1	41980420	41498180	97.33	95.38	43.45	3.52%	87.54%
Fm_2	46867074	46224772	97.31	95.36	43.52	3.43%	86.28%
Fm_3	44868636	44146756	97.15	95.14	42.98	3.01%	78.64%
Fm_4	44608142	44074456	97.19	95.23	43.47	3.27%	83.54%

#### 3.4.2. Analysis of DEGs

**Figure 4.**
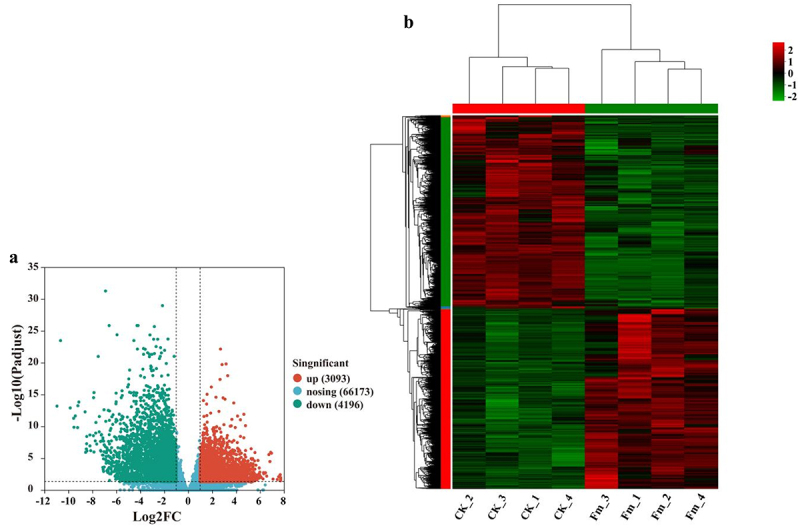
(a) volcano plot and (b) cluster heatmap of DEGs.

The DEGs between the Fm and CK groups were analyzed. A volcano plot (Figure 4a) identified a total of 8099 DEGs, with 3903 genes up-regulated and 4196 genes down-regulated. A cluster heatmap (Figure 4b) revealed the significant differences in the gene expression profiles between the Fm and CK groups (*p* < 0.05).

#### 3.4.3. Functional enrichment analysis

**Figure 5.**
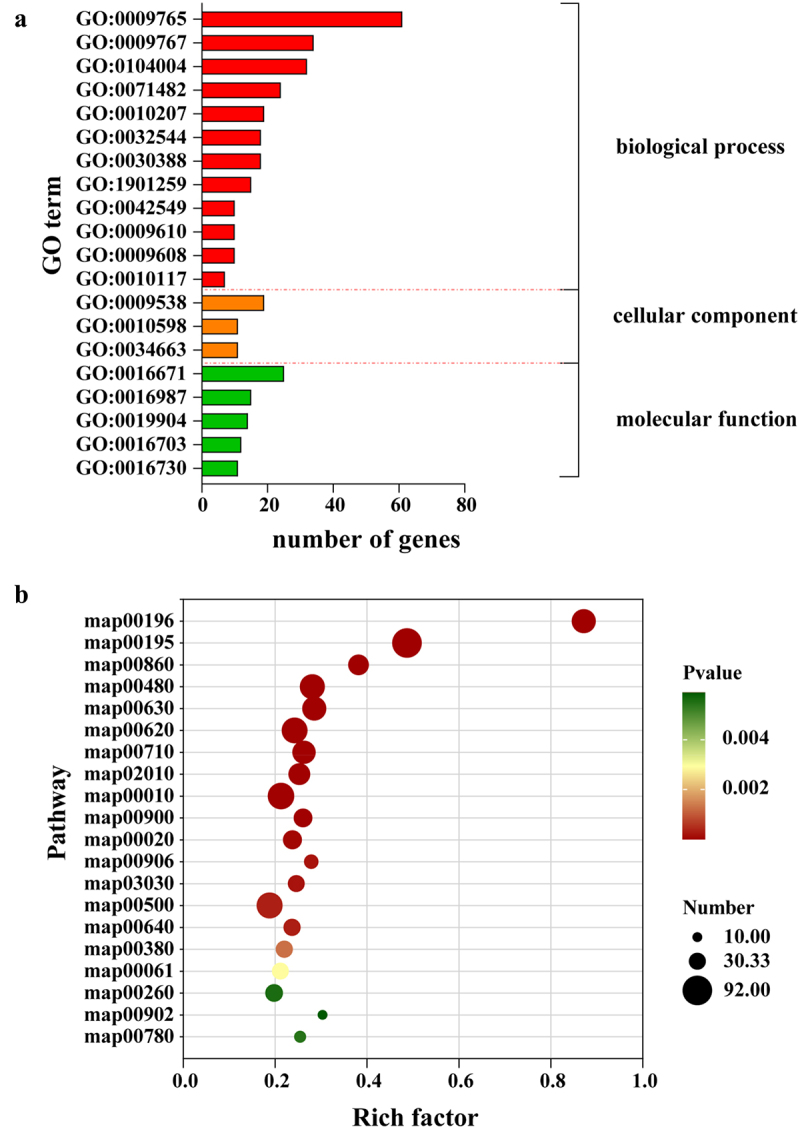
(a) GO and (b) KEGG enrichment analysis of DEGs.

The GO enrichment analysis was conducted on DEGs (Top 20 items). As shown in Figure 5a, in the molecular function category, DEGs were predominantly enriched for oxidoreductase activity, acting on the sulfur group of donors with disulfide as an acceptor (GO:0016671), sigma factor activity (GO:0016987), and protein domain-specific binding (GO:0019904). In the biological process category, DEGs were mainly enriched in photosynthesis, light harvesting (GO:0009765), photosynthetic electron transport chain (GO:0009767), and cellular response to environmental stimulus (GO:0104004). In the cellular component category, DEGs were primarily enriched in photosystem I reaction center (GO:0009538), endoplasmic reticulum chaperone complex (GO:0034663), and NAD(P)H dehydrogenase complex (plastoquinone) NAD(P)H (GO:0010598).

GO:0009765: photosynthesis, light harvesting, GO:0009767: photosynthetic electron transport chain, GO:0104004:cellular response to environmental stimulus, GO:0071482: cellular response to light stimulus, GO:0010207: photosystem II assembly, GO:0030388: fructose 1,6-bisphosphate metabolic process, GO:0032544: plastid translation, GO:1901259: chloroplast rRNA processing, GO:0009608: response to symbiont, GO:0009610: response to symbiotic fungus, GO:0042549: photosystem II stabilization, GO:0010117: photoprotection, GO:0009538: photosystem I reaction center, GO:0034663: endoplasmic reticulum chaperone complex, GO:0010598: NAD(P)H dehydrogenase complex (plastoquinone)NAD(P)H, GO:0016671: oxidoreductase activity, acting on a sulfur group of donors, disulfide as acceptor, GO:0016987: sigma factor activity, GO:0019904: protein domain specific binding, GO:0016703: oxidoreductase activity, acting on single donors with incorporation of molecular oxygen, incorporation of one atom of oxygen (internal monooxygenases or internal mixed function oxidases), GO:0016730: oxidoreductase activity, acting on iron-sulfur proteins as donors. B: map00196: Photosynthesis – antenna proteins, map00195: Photosynthesis, map00860: Porphyrin metabolism, map00480: Glutathione metabolism, map00630: Glyoxylate and dicarboxylate metabolism, map00620: Pyruvate metabolism, map00710: Carbon fixation in photosynthetic organisms, map02010: ABC transporters ABC, map00010: Glycolysis/Gluconeogenesis, map00900: Terpenoid backbone biosynthesis, map00020: Citrate cycle (TCA cycle), map00906: Carotenoid biosynthesis, map03030: DNA replication, map00500: Starch and sucrose metabolism, map00640: Propanoate metabolism, map00380: Tryptophan metabolism, map00061: Fatty acid biosynthesis, map00260: Glycine, serine and threonine metabolism, map00902: Monoterpenoid biosynthesis, map00780: Biotin metabolism.

The KEGG enrichment analysis was conducted on DEGs (Top 20 pathways). As shown in Figure 5b, DEGs were primarily enriched in the following pathways: photosynthesis – antenna proteins (map00196), photosynthesis (map00195), porphyrin metabolism (map00860), glutathione metabolism (map00480), glyoxylate and dicarboxylate metabolism (map00630), pyruvate metabolism (map00620), carbon fixation in photosynthetic organisms (map00710), ABC transporters (map02010), glycolysis/gluconeogenesis (map00010), terpenoid backbone biosynthesis (map00900), and citrate cycle (TCA cycle) (map00020).

Overall, the DEGs between Fm and CK were predominantly enriched in pathways associated with the biological process, molecular function, and metabolism. Integrating the results from physiological growth indicators with the RNA-Seq data, this study focused on the expression of DEGs related to growth promotion, antioxidase, and terpenoid backbone biosynthesis.

#### 3.4.4. Analysis of DEGs related to growth promotion

**Figure 6.**
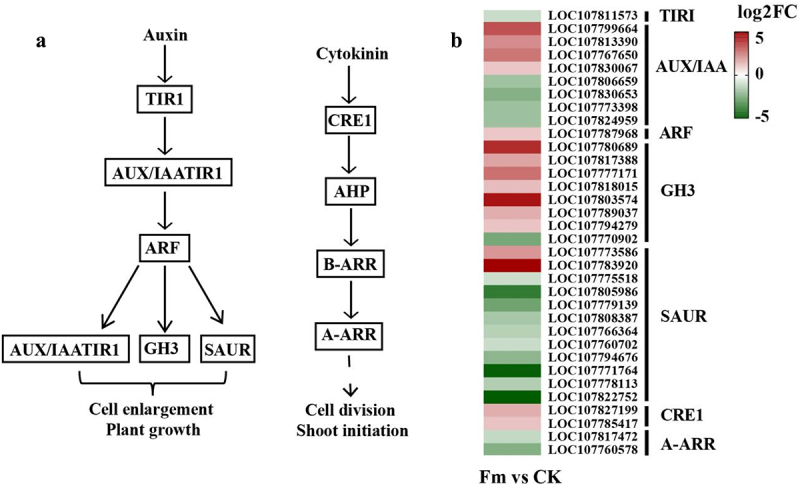
Auxin and cytokinin involvement in (a) major pathways and (b) changes in the expression of key genes.

TIR1: transport inhibitor response 1; AUX1: auxin influx carrier; IAA: auxin-responsive protein IAA; ARF: auxin response factor; GH3: auxin responsive GH3 gene family; SAUR: SAUR family protein; CRE1: uncharacterized; A-ARR: two-component response regulator ARR

Compared with the CK group, 30 DEGs related to the auxin signal transduction were identified in the Fm group (Figure 6). Notably, 14 were up-regulated, including 4 involved in coding AUX/IAA (*LOC107799664*, *LOC107813390*, *LOC107767650*, and *LOC107830067*), 1 coding ARF (*LOC107787968*), 7 coding GH3 (*LOC107780689*, *LOC107817388*, *LOC107777171*, *LOC107818015*, *LOC107803574*, *LOC107789037*, and *LOC107794279*), and 2 coding SAUR *(LOC107773586* and *LOC107783920*). Additionally, 16 DEGs were down-regulated, including 1 coding TIRI (*LOC107811573*), 4 coding AUX/IAA (*LOC107806659*, *LOC107830653*, *LOC107773398*, and *LOC107824959*), 1 coding GH3 (*LOC107770902*), and 10 coding SAUR (*LOC107779139*, *LOC107794676*, *LOC107775518*, *LOC107808387*, *LOC107760702*, *LOC107822752*, *LOC107778113*, *LOC107771764*, *LOC107766364*, and *LOC107805986*). All these DEGs indirectly regulated cell expansion and plant growth.

Compared with the CK group, four DEGs related to the cytokinin signal transduction were identified in the Fm group (Figure 6). Among these, two DEGs coding for CRE1 (*LOC107827199* and *LOC107785417*) were up-regulated, while two DEGs coding for A-ARR *(LOC107817472* and *LOC107760578*) were down-regulated. All these DEGs indirectly regulated cell division and shoot growth.

#### 3.4.5. Analysis of key genes related to antioxidase

**Figure 7.**
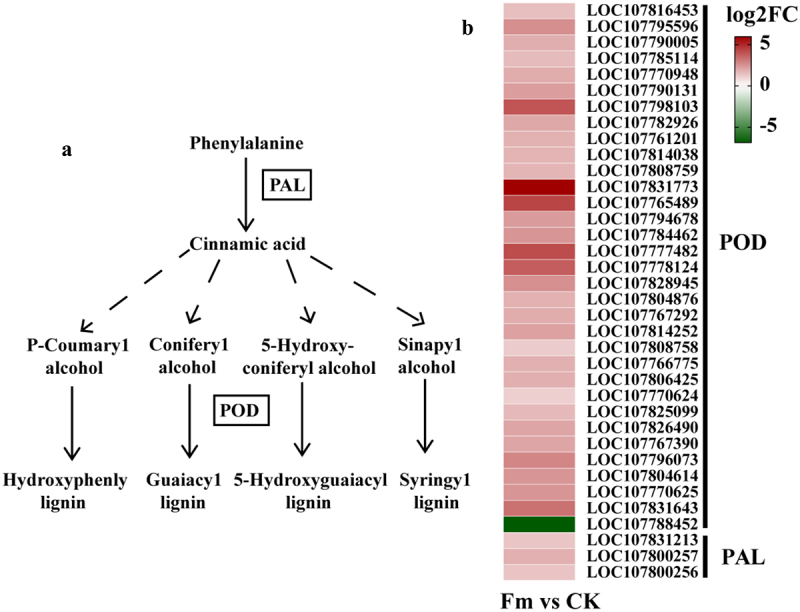
PAL and POD (a) involvement in major pathways and (b) changes in the expression of key genes.

PAL: phenylalanine ammonia-lyase; POD: peroxidase

Compared to the CK group, 37 DEGs related to antioxidase were identified in the Fm group (Figure 7). Among these, three DEGs (*LOC107831213*, *LOC107800257*, and *LOC107800256*) involved in regulating PAL for the cinnamic acid synthesis were up-regulated. Additionally, 32 DEGs (*LOC107816453*, *LOC107795596*, *LOC107790005*, *LOC107785114*, *LOC107770948*, *LOC107790131*, *LOC107798103*, *LOC107782926*, *LOC107761201*, *LOC107814038*, *LOC107808759*, *LOC107831773*, *LOC107765489*, *LOC107794678*, *LOC107784462*, *LOC107777482*, *LOC107778124*, *LOC107828945*, *LOC107804876*, *LOC107767292*, *LOC107814252*, *LOC107808758*, *LOC107766775*, *LOC107806425*, *LOC107770624*, *LOC107825099*, *LOC107826490*, *LOC107767390*, *LOC107796073*, *LOC107804614*, *LOC107770625*, and *LOC107831643*) and 1 DEG (*LOC107788452*) involved in regulating POD for the synthesis of P-hydroxyphenyl lignin, guaiacyl lignin, 5-hydroxyguaiacyl lignin, and syringyl lignin were up-regulated and down-regulated, respectively.

#### 3.4.6. Terpenoid backbone biosynthesis

The KEGG enrichment analysis revealed that 37 DEGs were annotated into the terpenoid backbone biosynthesis pathway, with 30 key genes involved in the carotenoid biosynthesis regulating 15 key enzymes (Table 6). Specifically, the MVA pathway included 15 genes regulating 7 enzymes: two up-regulated genes for the acetyl-CoA acetyltransferase, four up-regulated genes for the hydroxymethylglutaryl-CoA synthase-like, two up-regulated genes for 3-hydroxy-3-methylglutaryl-coenzyme A reductase 1, two up-regulated genes for the mevalonate kinase-like, two up-regulated genes for phosphomevalonate kinase-like, two up-regulated genes for diphosphomevalonate decarboxylase MVD2-like, and one up-regulated gene for isopentenyl phosphate kinase (ipk). The MEP pathway included six genes regulating four enzymes: two down-regulated genes for the 1-deoxy-D-xylulose-5-phosphate synthase, one down-regulated gene for the 1-deoxy-D-xylulose 5-phosphate reductoisomerase, two down-regulated genes for the 4-hydroxy-3-methylbut-2-enyl diphosphate reductase, and one up-regulated gene for the isopentenyl-diphosphate Delta-isomerase I-like. In addition, the remaining genes involved nine genes regulating four enzymes: one up-regulated and one down-regulated gene for the geranylgeranyl pyrophosphate synthase, four up-regulated genes for the farnesyl pyrophosphate synthase 1-like, one up-regulated gene for the probable phytol kinase 2, chloroplastic, and one up-regulated and one down-regulated gene for PCME.

**Table 6.** DEGs related to carotenoid biosynthesis in tobacco rhizomes and log2fc.

EC	gene name	gene description	FmvsCK(log2fc)
2.3.1.9	*LOC107765596*	acetyl-CoA acetyltransferase, cytosolic 1	2.24
	*LOC107819092*	acetyl-CoA acetyltransferase, cytosolic 1-like	2.10
2.3.3.10	*LOC107796407*	hydroxymethylglutaryl-CoA synthase-like	2.34
	*LOC107798568*	hydroxymethylglutaryl-CoA synthase-like	1.50
	*LOC107805038*	hydroxymethylglutaryl-CoA synthase-like	2.18
	*LOC107816507*	hydroxymethylglutaryl-CoA synthase-like	2.37
1.1.1.34	*LOC107772124*	3-hydroxy-3-methylglutaryl-coenzyme A reductase 1	1.06
	*LOC107759218*	3-hydroxy-3-methylglutaryl-coenzyme A reductase 1-like	1.49
2.7.1.36	*LOC107815397*	mevalonate kinase-like	1.37
	*LOC107829822*	mevalonate kinase-like	1.36
2.7.4.2	*LOC107823984*	phosphomevalonate kinase-like	1.49
	*LOC107791244*	phosphomevalonate kinase-like	1.38
4.1.1.33	*LOC107817230*	diphosphomevalonate decarboxylase MVD2-like	1.62
	*LOC107811776*	diphosphomevalonate decarboxylase MVD2-like	2.12
2.7.4.26	*LOC107793526*	ipk	1.17
2.2.1.7	*LOC107790348*	probable 1-deoxy-D-xylulose-5-phosphate synthase, chloroplastic	−1.08
	*LOC107818796*	probable 1-deoxy-D-xylulose-5-phosphate synthase, chloroplastic	−1.14
1.1.1.267	*LOC107818871*	1-deoxy-D-xylulose 5-phosphate reductoisomerase, chloroplastic	1.24
1.17.7.4	*LOC107760258*	4-hydroxy-3-methylbut-2-enyl diphosphate reductase, chloroplastic-like	−1.14
	*LOC107765472*	4-hydroxy-3-methylbut-2-enyl diphosphate reductase, chloroplastic-like	−1.23
5.3.3.2	*LOC107808635*	isopentenyl-diphosphate Delta-isomerase I-like	1.57
2.5.1.1/2.5.1.29	*LOC107770520*	heterodimeric geranylgeranyl pyrophosphate synthase small subunit, chloroplastic-like, transcript variant X1	−1.02
	*LOC107822513*	geranylgeranyl pyrophosphate synthase 7, chloroplastic-like	3.07
2.5.1.1/2.5.1.10	*LOC107779968*	farnesyl pyrophosphate synthase 1-like	2.05
	*LOC107806551*	farnesyl pyrophosphate synthase 1-like	2.58
	*LOC107795792*	farnesyl pyrophosphate synthase 1-like	1.98
	*LOC107765018*	farnesyl pyrophosphate synthase 1-like	1.79
2.7.1.216	*LOC107798184*	probable phytol kinase 2, chloroplastic	4.18
PCME	*LOC107829081*	probable isoprenylcysteine alpha-carbonyl methylesterase ICMEL2	−3.02
	*LOC107792450*	probable isoprenylcysteine alpha-carbonyl methylesterase ICMEL2	1.55

**Figure 8.**
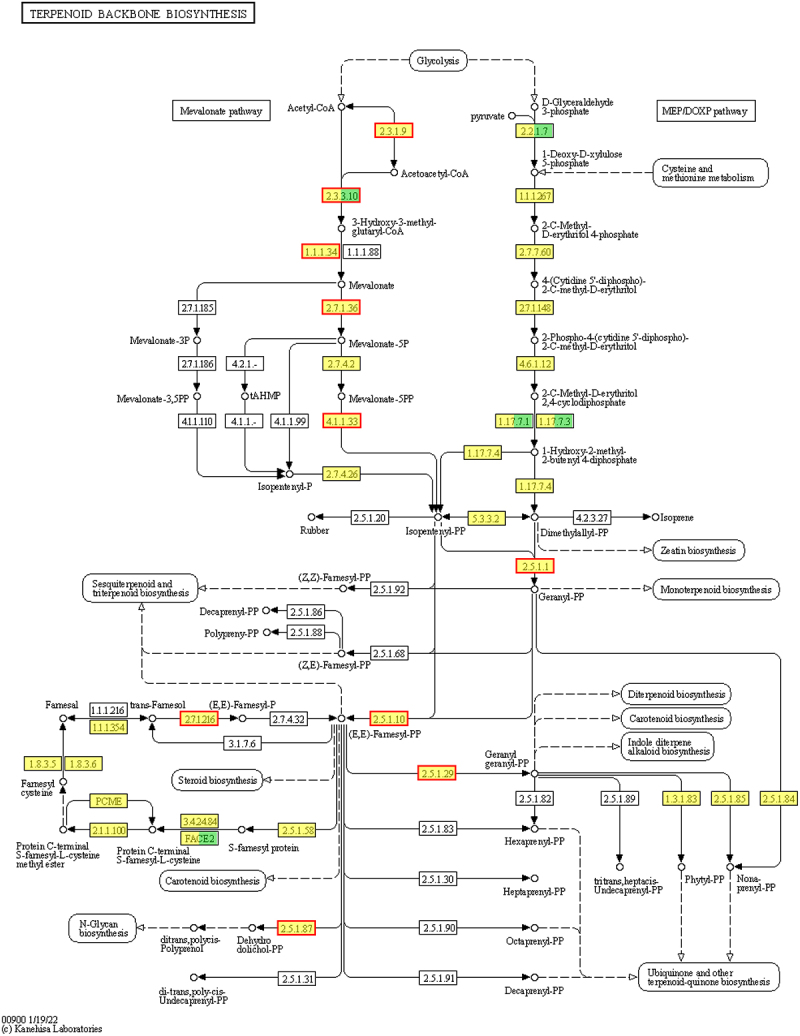
Terpenoid backbone biosynthesis pathway.

In tobacco rhizomes, the final products of both the MVA and MEP pathways are isopentenyl-PP (IPP) and its isomer dimethylallyl-PP (DPP). Both IPP and DPP produce geranyl-PP (GPP) through the action of geranylgeranyl pyrophosphate synthase 7 and chloroplastic-like enzymes. The GPP is converted to the farnesyl-PP (FPP) via the farnesyl pyrophosphate synthase 1-like. Furthermore, PCME and phytol kinase 2 are likely to contribute to the production of FPP through alternative pathways. FPP is also converted to the geranylgeranyl-PP (GGPP) via geranylgeranyl pyrophosphate synthase. Both FPP and GGPP are the key substances involved in the biosynthesis of carotenoids (Figure 8).

#### 3.4.7. Verification by q-PCR

**Figure 9.**
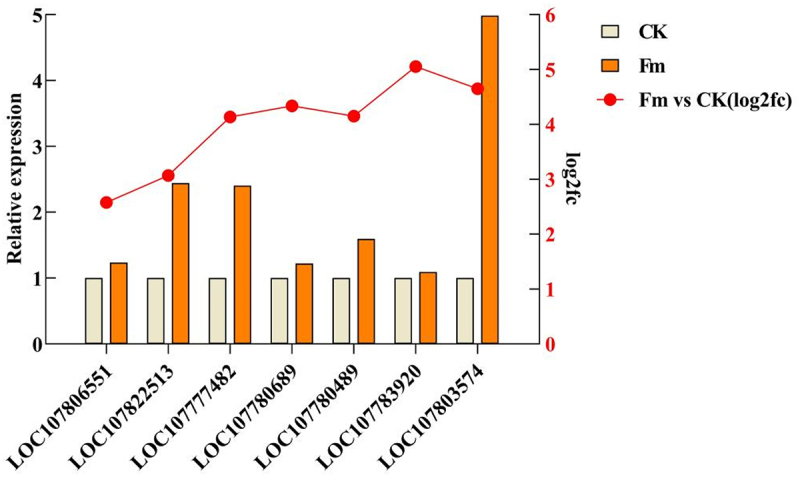
RNA-Seq and qRT-pcr gene expression results.

The accuracy and reproducibility of the RNA-Seq data were confirmed through the qRT-PCR validation using actin as the internal reference gene. Seven key DEGs related to the growth promotion, the antioxidase activity, and the terpenoid backbone biosynthesis were selected for validation. The results of qRT-PCR were highly in accordance with the trend of alterations in the transcriptome data. The expression levels of the 7 DEGs genes were all upregulated upon the induction of Fm treatment. Compared with the control group, Fm treatment elevated the expression of *LOC107806551*, *LOC107822513*, *LOC107777482*, *LOC107780689*, *LOC107780489*, *LOC107783920*, and *LOC107803574* by 1.24, 2.44, 2.41, 1.22, 1.59, 1.09, and 4.97 times respectively, thereby confirming the reliability of the transcriptome sequencing results (Figure 9).

The broken line is the gene expression analyzed by RNA-Seq, and the bar is the relative gene expression determined by qRT-PCR.

## 4. Discussion

The growth-promoting effect of AMF on plants is closely related to the extent of mycorrhizal colonization between AMF and host plants. The mycorrhizal colonization rate serves as a key indicator for assessing the development of AMF-infected plant roots, including mycelia, arbuscular branches, and vesicles. The higher colonization rate indicates a greater degree of mycorrhizalization, which benefits plant growth.^26^ In this study, Ab, Sv, Fm, Ce, Ri, and H all invaded the tobacco roots and established symbiosis. Notably, the Fm group exhibited the highest colonization rate, root mycorrhizal infection density, arbuscular abundance, and spore density. Zhang et al.^27^ examined the symbiotic relationship between tomato plants and three AMF species *(R. intraradices*, *F. mosseae*, and *R. irregularis*) under both well-watered and drought conditions. Their findings indicated that all three AMF species were capable of colonizing tomato roots, which aligns closely with the results of this study. Furthermore, compared with the CK group, the growth indicators, such as plant height, root length, leaf area, stem diameter, and biomass, were increased to the varying degrees in all AMF treatments, except for the H group, with slightly lower root length and stem diameter. This finding aligned with those of previous studies.^28–30^ Among the five AMF treatments, Fm and Sv exhibited significantly higher growth indicators than the CK group. Specifically, the Fm group had the highest plant height, below-ground biomass, and total biomass, whereas the Sv group had the highest root length, leaf area, stem diameter, and above-ground biomass. Navarro and Morte^31^ observed that both Ri and Fm could effectively infect the lime roots and significantly enhance the growth, with Ri presenting the greater growth-promoting effect than Fm. Thus, the effectiveness of AMF in promoting plant growth varied with plant species and AMF types, reflecting their selective affinity and functional roles.

Antioxidase plays a crucial role in plants as a protective mechanism against the reactive oxygen species (ROS) and is typically positively correlated with the plant growth and the stress resistance.^32^ In this study, the AMF inoculation enhanced the activities and contents of SOD, POD, PPO, and PAL in tobacco leaves, with the varying effects depending on the microbial agent. Specifically, the Sv group exhibited the highest SOD, POD, and PPO activities, whereas the Ri group had the highest PAL activity. Concurrently, all the AMF treatments increased the tobacco chlorophyll, IAA, CTK, the soluble sugar, proline, the total phenol, and the flavonoid content, and reduced the MDA content. Zhou et al.^33^ also observed that the Fm inoculation increased the soluble sugar content and the POD and SOD activities, while decreasing the MDA content in tomato fruits under the saline-alkaline stress. Cui et al.^28^ demonstrated that the inoculation with Fm, irrespective of exogenous calcium ions (Ca^2+^), enhanced the chlorophyll content, the antioxidase activity, the total phenol, and the flavonoid contents in peanut leaves and roots, while reducing the MDA content. This indicated that AMF could promote the antioxidase activity in plants, increase the content of chlorophyll, IAA, CTK and osmotic substances such as soluble sugars, and phenols, enhance the photosynthesis, and promote the plant growth. Additionally, the comprehensive evaluation of the membership function revealed that various AMF microbial agents positively affected the tobacco growth. Among them, the Fm group exhibited the highest membership function, indicating that it had the most significant growth promotion effect. Thus, the efficacy of different AMF microbial agents on tobacco growth varied, likely because of the specific interactions between AMF and host plants. Sharma and Sharma^34^ identified significant variations in the functional attributes and root-infection capabilities among different AMF species. These differences may underlie the varying regulatory effects of AMF on plant enzymes, soluble sugars, phenolic compounds, and other osmotic substances, ultimately leading to disparities in their growth-promoting effects.

To further elucidate the changes in key gene expression profiles associated with Fm-induced growth promotion in tobacco, RNA-Seq was conducted on the tobacco rhizomes of the CK and Fm groups, resulting in the identification of 8099 DEGs. The GO enrichment analysis revealed that, compared to the CK group, the DEGs in the Fm group were predominantly related to biological processes, molecular functions, and cellular components. The KEGG enrichment analysis indicated that these DEGs were primarily involved in pathways such as photosynthesis-antenna proteins, Photosynthesis, Porphyrin metabolism, and glutathione metabolism. Liu et al.^35^ reported the similar findings, where DEGs in Amorpha fruticosa roots, induced by Fm, were also annotated in pathways related to biological processes, molecular functions, and metabolism, aligning with our study results. Moreover, the differential expression was observed in genes that regulated the synthesis of auxin, cytokinin, antioxidase, and carotenoids, which were crucial for tobacco growth. This information could provide valuable insights into the key genes involved in the AMF-induced growth promotion in tobacco and offer a reference for the development and application of AMF as growth promoters.

When plants receive specific external signals, their local tissues can synthesize trace amounts of hormones that bind to specific protein receptors.^35^ The auxin and cytokinin, as two of the most widely distributed and complex plant hormones, could regulate downstream responsive gene expression through signaling pathways to enhance plant growth and development.^36^ In this study, the Fm group exhibited a significant enrichment of 30 DEGs related to the auxin signal transduction compared to the CK group. Of these, 4 DEGs involved in coding AUX/IAA, 1 in coding ARF, 7 in coding GH3, and 2 in coding SAUR were up-regulated, while 1 DEG in coding TIRI, 4 in coding AUX/IAA, 1 in coding GH3, and 10 in coding SAUR were down-regulated. Aux/IAA, GH3, and SAUR are primary auxin-responsive genes, whereas ARFs are transcription factors that bind to auxin-responsive elements (AuxREs) to regulate the expression of auxin-responsive genes.^37,38^ Aux/IAA has been shown to bind to the auxin-responsive factor ARF, regulating the expression of auxin-encoding genes and promoting oat growth and development.^39^ In addition, compared with the CK group, the tobacco in the Fm group exhibited the significant enrichment of four DEGs related to the cytokinin signal transduction. Specifically, two DEGs involved in coding CRE1 were up-regulated, and two DEGs involved in coding A-ARR were down-regulated. CRE1, a cytokinin receptor, self-phosphorylates upon receiving cytokinin signals and transmits these signals to A-ARRs in the nucleus through Arabidopsis His phosphotransfer proteins (AHPs) to regulate plant growth.^40,41^ A-ARRs that possess the signal-receiving domains but not signal-output domains play a negative regulatory role in the cytokinin signal transduction.^42^ Therefore, Fm can induce the changes in hormone levels by stimulating the expression of genes related to hormone biosynthesis and signal transduction, thereby promoting tobacco growth.^43^

Antioxidase plays a crucial role in plant growth and development by promoting cell elongation, increasing lignin production, clearing ROS, and enhancing stress resistance.^44^ In this study, the Fm group exhibited a significant enrichment of 37 DEGs related to antioxidase compared to the CK group. Among these, three up-regulated DEGs influenced the cinnamic acid biosynthesis by regulating the PAL activity, whereas 32 up-regulated DEGs and 1 down-regulated gene affected lignin biosynthesis by regulating the POD activity. Specifically, L-phenylalanine is converted to cinnamic acid by PAL, and increased POD activity promotes the formation of lignin polymers through the spontaneous coupling of monophenolic radicals in tomato cells.^45^ These findings can align with the results of this study, highlighting the importance of cinnamic acid and lignin as key products of the phenylpropane biosynthesis pathway. The cinnamic acid is a self-toxic substance released by plants through leaching, residue decomposition, and root secretion, which promotes plant growth at low concentrations but inhibits growth at high concentrations.^46^ Lignin, an aromatic polymer formed from the polymerization of three monomers, constitutes approximately 15% to 36% of the total plant biomass and features a compact molecular structure. As a crucial component of the plant cell wall, lignin strengthens the cell wall through reticular intertwining, supports the plant growth, and influences the overall plant development.^47^ Therefore, POD and PAL are the essential enzymes for promoting the tobacco growth through Fm, and future studies should focus on the key genes that encode these enzymes.

Tetraterpenoids, comprising eight isoprene units and containing 40 carbon atoms, are characterized by a series of conjugated double-bond chromophores, which impart color and contribute to their lipophilic nature.^48^ Carotenoids, significant tetraterpenoids, absorb the light energy and transfer it to chlorophyll for photosynthesis, thereby promoting plant growth.^49^ FPP that can be synthesized via the MVA pathway from acetyl coenzyme A and the MEP pathway from pyruvate and 3-phosphoglyceraldehyde plays a crucial role in carotenoid synthesis.^50^ This study identified 15 enzymes, including ACAT, HMGCS, and MVK, involved in FPP synthesis in tobacco rhizomes, with 13 of these enzymes aligned with those reported by Majer et al.^50^ for the tobacco carotenoid biosynthesis. Additionally, a total of 30 DEGs related to carotenoid biosynthesis were identified in the Fm group compared to the CK group, with most DEGs up-regulated and a small proportion down-regulated. Consequently, Fm enhances carotenoid biosynthesis by regulating genes associated with MVA, MEP, and related pathways, thereby promoting photosynthesis and improving tobacco growth.

## 5. Conclusion

The study conclusively demonstrated that all the examined strains of AMF effectively formed the symbiotic relationships with tobacco, significantly enhancing the plant growth. Each microbial agent increased the enzymatic activities of SOD, POD, PPO, and PAL in tobacco. Moreover, they elevated the levels of chlorophyll, IAA, CTK, soluble sugars, and proline, while reducing MDA, a marker of oxidative stress.

*Funneliformis mosseae* (Fm) emerged as the most effective AMF strain to promote the tobacco growth. The RNA-sequencing analysis indicated that compared to the control group (CK), the tobacco rhizome of the Fm group exhibited 8099 DEGs. Most of these DEGs were related to essential biological processes, molecular functions, and metabolic pathways, as demonstrated by GO and KEGG functional enrichment analyses.

Additionally, the study pinpointed the differential expression of genes implicated in the synthesis of auxin and cytokinin, as well as those regulating the antioxidant activity and the carotenoid biosynthesis in the tobacco rhizome of the Fm group. These findings suggested that AMF, particularly Fm, primarily promoted the tobacco growth by modulating the auxin levels, enhancing the antioxidase activity, and regulating the carotenoid biosynthesis.

## Data Availability

No data was used for the research described in the article.
